# The relationship between the use of mental health act and general population suicide rates in England and Wales

**DOI:** 10.5249/jivr.v4i1.66

**Published:** 2012-01

**Authors:** Ajit Shah

**Affiliations:** ^a^University of Central Lancashire, Preston, United Kingdom and Consultant Psychiatrist, West London Mental Health NHS Trust, London, United Kingdom.

## Abstract

**Background:**

The relationship between suicide and involuntary admissions has been mainly examined in younger and mixed age groups. These studies provide mixed results with some demonstrating no relationship and others reporting increased rates of suicides in involuntarily admitted patients. However, the association between the utility of the Mental Health Act with general population suicide rates in England and Wales has not been formally studied.

**Methods:**

Thus, an ecological study, over the 19-year period, to examine the relationship between rates of involuntary admissions and general population suicide rates in England and Wales was undertaken using nationally collected data. Data on general population suicide rates for both sexes were ascertained from the World Health Organization (WHO) website. Data on the number of detentions under the Mental Health Act were ascertained from the Office of National Statistics website. Data on the population size for the elderly age-bands were ascertained from the WHO website. Spearman’s correlation coefficient was used to examine the relationship between suicide rates and rates of detention under the Mental Health Act.

**Results:**

There were negative correlations between rates of involuntary admissions and general population suicide rates in both sexes.

**Conclusions:**

A causal relationship and the direction of causality cannot be assumed because this was an ecological study. There is a need for sufficiently powered study to compare the number of suicide occurring in involuntarily and voluntarily admitted patients using a case-control or cohort design and survival analysis. If an inverse association can be demonstrated between suicide and involuntary admissions then it has important implications for the development of mental health legislation as an adjunct to national suicide prevention strategies.

## Introduction

Involuntary admission into hospital under relevant mental health legislation is generally used to protect patients or others from harm. There has been Mental Health Legislation in England and Wales since the 19th century. Mental Health Act 1983 will be examined in in this study, although it has a precursor (Mental Health Act 1959). The Mental Health Act in England and Wales recommends that patients should generally be admitted under Section 2 (for assessment lasting 28 days) or Section 3 (for treatment lasting 6 months) of the Act, which requires two doctors and an approved mental health professional (e.g. a social worker) to complete. Section 4 can only be justified in emergencies when only one doctor is available, it lasts for only 72 hours, and either a Section 2 or Section 3 must be completed during this 72 hour period. Part III of the Mental Health Act applies to patients who have been alleged to have committed a crime and their detention in hospital emerges through the criminal justice system. Essentially detention in hospital under this Mental Health Act is considered if the patient’s health or safety are at risk or the safety of others is at risk. In this context, individuals at potential risk of suicide may be involuntarily admitted.^1^Moreover, there is increased risk of suicide within the first seven days of involuntary hospital admission.^2^

A cross-national and ecological study of 100 countries reported that general population suicide rates increased after the introduction of mental health legislation.^3^Increased awareness of both mental health issues and presence of mental health legislation may lead to greater use of the mental health legislation leading to treatment of mental illness and possible reduction in the risk of suicide. Both involuntary and voluntary admissions may reduce the risk of suicide, but it is possible that those at the highest risk of suicide may be admitted involuntarily. However, in another cross-national and ecological study, there was no significant association between presence of mental health legislation and elderly suicide rates.^4^Yet the mere presence of mental health legislation may not necessarily lead to consistent implementation of this legislation, and this may explain an absence of association between decline in suicide rates and implementation of mental health legislation in both these studies.^3,4^Moreover, the criteria for involuntary admission and the actual process of involuntary admission varies across countries.

A Canadian study comparing the number of suicides between those with involuntary and voluntary psychiatric admissions at nine year follow-up found no difference between the two groups.^5^A study of suicides within one year of contact with mental health services in England and Wales reported an increase in the number of suicides in those aged under 25 years admitted involuntarily.^6^However, this association was not observed in other English studies of psychiatric inpatients discharged and followedup for one year^7^and two to twelve years.^8^Another English study reported that the number of suicides during the admission were significantly higher in those involuntarily than voluntarily admitted,^9^but this may reflect severity of illness requiring admission.^9^A recent ecological study demonstrated a negative correlation between elderly suicide rates and rates of use of the mental health act in England and Wales.^10^Comparison between these different studies are difficult because of the different methodologies used and different criteria for the use of mental health legislation and different processes to implement it.

The association of the Mental Health Act with general population suicide rates in England and Wales has not been formally studied. Thus, an ecological study, over a 19-year period, to examine the relationship between rates of involuntary admissions and general population suicide rates in England and Wales was undertaken. Given the mixed findings of an association in the literature, a null hypothesis that there will be no association between rates of involuntary admissions and general population suicide rates was proposed.

## Methods

Data on general population suicide rates for males and females for England and Wales was ascertained from the World Health Organisation (WHO) website (http://www.who.int/whosis/database/mort/table1.cfm) for each of the 19 years between 1988 and 2006.

Data on the number of detentions under the Mental Health Act 1983 (MHA1983) for all detentions, those under Sections 4, 2 and 3 of the MHA1983 and those under Part III of the MHA1983, were ascertained from the Office of National Statitistics website (http://www.dh. gov.uk/en/Publicationsandstatistics/Statistics/StatisticalWorkAreas/Statisticalhealthcare/DH_4086494) for each of the 19 years between 1988 and 2006.

Data on the size of the general population in England and Wales were also ascertained from the WHO website (http://www.who.int/whosis/database/mort/table1.cfm) for each of the 19 years between 1988 and 2006.

The rates of detention under the MHA1983 for all detentions, detentions under Sections 4, 2 and 3 of the MHA1983, and detentions under Part III of the MHA1983 were calculated by dividing the number of detentions in each category by the size of the general population for each of the 19 years.

The relationship between general population suicide rates in both sexes and the rates of detention under the MHA1983 for each category of detention across the 19 years was examined with Spearman’s correlation coefficient (rho) because data on both suicide rates and rates of detention under the Mental Health Act were not normally distirbuted. A similar methodology was used in a study of elderly suicides.^10^

## Results

Full data sets were available for the 19-year period 1988 to 2006. The median (range) of general population suicide rates over the 19-year period in males and females were 10.9 (9.6-12.7) and 3 (2.8-4.5) per 100,000 population respectively. The median (range) of the number of detentions over the 19-year period for all detentions, Section 2 detentions, Section 3 detentions and Section 4 detentions were 25642 (15993-27716), 13175 (9515-15268), 8968 (2887-9992) and 1581 (1051-2048) resepctively. Table 1illustrates the relationship between general population suicide rates in both sexes and the rates of detention under the MHA1983 for each category of detention. There was no significant correlation between general population suicide rates in both sexes and rates of detention under Section 4.

**Table:** Table 1: **The relationship between general population suicide rates and rates of detention under the Mental Health Act 1983 in England and Wales**

RATES OF DETENTION(under Mental Health Act)	GENERAL POPULATION SUICIDE RATES	
	Males	Females
All Detentions	Rho=-0.67	Rho=-0.51
	P=0.002	P=0.027
Section 4	NS	NS
Section 2	Rho=-0.76	Rho=-0.53
	P<0.0001	P=0.02
Section 3	Rho=-0.55	Rho=-0.46
	P=0.015	P=0.048
Detention under Part III of MHA1983	Rho=-0.54	NS
	P=0.018	

NS= Not Significant Rho= Spearman’s correlation coefficient.

There were highly significant negative correlations between general population suicide rates in both sexes and rates of detention for all detentions and those under Sections 2 and 3 of the MHA1983. There was a siginificant negative correlation between general population suicide rates in males and detention under Part III of the Mental Health Act, but this was not observed in females.

## Discussion

There was no association between general population suicide rates and involuntary admission under Section 4 of the Mental Health Act. The Mental Health Act in England and Wales recommends that patients should generally be admitted under Section 2 or Section 3 of the Act, which requires two doctors and an approved mental health professional (e.g. a social worker) to complete, and where possible to avoid involuntary admission under Section 4. Section 4 can only be justified in emergencies when only one doctor is available, it lasts for only 72 hours, and either a Section 2 or Section 3 must be completed during this 72 hour period. Consequently Section 4 is infrequently used, and this limited use may explain an absence of association with suicide rates.

There were negative correlations between general population suicide rates in both sexes and the overall rates of involuntary admission and rates of involuntary admission under Sections 2 and 3 of the Mental Health Act. These findings are consistent with those reported for younger and mixed age groups in some studies^6,8^and in one identical study of elderly suicides.^10^ However, they were not consistent with opposite findings in one study^3^and the absence of an association in other studies,^5,7,8^including a cross-national study of elderly suicides.^4^

The observed correlations in the current study may be due to methodological issues. In England and Wales, the coroner can only return a verdict of suicide if suicide can be proved beyond reasonable doubt. Therefore, some genuine suicides may be misclassified as an open verdict when suicide cannot be proved beyond a reasonable doubt.^11^ This may have resulted in a lower genuine general population suicide rate being included in the analysis, but data on deaths due to open verdicts were not available from the WHO for the period before 2001 when the ICD-9 classification was used. It is also posible that other factors may independently influence elderly suicide rates and rates of involuntary admission in opposite directions leading to spurious correlations (epiphenomena). However, it is also possible that the correlations are genuine and this is examined below.

If the results are genuine then there are two possibilities: first, reduced general population suicide rates may lead to an increase in rates of involuntary admissions; and second, increased rates of involuntary admission may reduce general population suicide rates. There is little theoretical, clinical or legal evidence to explain why lower general population suicide rates would lead to an increase in rates of involuntary admissions. A better case can be made to explain why increased rates of involuntary admission may lead to lower general population suicide rates. Rates of involuntary admissions may increase through several mechanisms. First, changes in mental health legislation may increase rates of involuntary admissions,^12^but this had not occured during the study period. Second, a range of national initiatives designed to reduce suicide rates may lead to an increase in rates of involuntary admissions, and these included: the Defeat Depression Campaign organised by the Royal College of Psychiatrist; the National Confidential Enquiry into Suicides and Homicides; the governmental “Our Healthier Nation” suicide reduction targets; and the National Suicide Prevention Strategy. There is evidence that these initiatives reduced elderly suicide rates.^13-15^Third, high profile investigation into suicides and high profile media reporting of suicides may also encourage clinicians to use mental health legislation for involuntary admissions more frequently. Fourth, increased rates of suicide may also encourage clinicians in increasing the use of mental health legislation for involuntary admissions; there is evidence of an association between mental health programmes and policy and increased suicide rates in the general population.^3^Increase in the rates of involuntary admisisons is likely to afford close supervision and protection of suicidal patients during the admission and facilitate reduction in the number of suicides. Moreover, treatment in hospital given to these involuntarily admitted patients may also lead to improvement in their mental illness and further facilitate reduction in the number of suicides.

However, a causal relationship and the direction of causality cannot be assumed because this was an ecological study. There is, therefore, a need for sufficiently powered study to compare the numbers of suicide occuring in involuntarily and voluntarily admitted patients, over at least a ten year follow-up period, using a case-control or cohort design and survival analysis. If an inverse association can be demonstrated between suicide and involutary admissions then this has important implications for the development of mental health legislation as an adjunct to national suicide prevention strategies.
